# Children’s and parents’ attitudes to and knowledge about HPV vaccination following a targeted information intervention

**DOI:** 10.1177/13674935241272004

**Published:** 2024-09-27

**Authors:** Eva Runngren, Karin Blomberg, Lina Schollin Ask, Emma Appelqvist, Madelene Danielsson, Mats Eriksson

**Affiliations:** 1 Faculty of Medicine and Health, School of Health Sciences, 6233 Örebro University, Örebro, Sweden; 2 25545 Public Health Agency of Sweden, Unit of vaccines, Solna, Sweden; 3 Department of Women’s and Children’s Health, 27106 Karolinska Institute, Solna, Sweden; 4 Department of Public Health Analysis and Data Management, 25545 Public Health Agency of Sweden, Solna, Sweden; 5 Department of Translational Medicine, Faculty of Medicine, Clinical Microbiology, 5193 Lund University, Lund, Sweden

**Keywords:** Attitude, child, human papillomavirus viruses, knowledge, parents

## Abstract

The aim of this study was to investigate Swedish children’s and parents’ attitudes and knowledge about human papillomavirus (HPV) vaccination a year after gender-neutral HPV vaccination was introduced in Sweden’s national immunization program (NIP). Additional information about HPV and vaccine was provided in the extended immunazation program. In total, 276 parents and 206 children from 22 School Health Services responded to a web-based survey. Results showed that half of the children and about a third of the parents received additional Public Health Agency information about HPV vaccination, and a majority were satisfied. Parents considered HPV vaccination being important for their children’s health, and both children and parents considered it important to vaccinate all genders against HPV. Both children and parents rated school nurses as most reliable source of HPV vaccination information. Teachers were also a common source of HPV and HPV vaccination information for children. Further research among teachers in Sweden is needed to explore their knowledge and abilities to inform students and parents about HPV and vaccination.

## Introduction

Cancer due to human papillomavirus (HPV) is a global cause of mortality. Worldwide about 342 000 women die each year from cervical cancer (World Health Organization, 2022). In Sweden, around 150 women die of cervical cancer each year (Public Health Agency of Sweden, 2022). Efficient vaccines are available to prevent diseases and infections caused by HPV (Castle and Maza, 2016), and knowledge about these vaccines is a crucial preventive tool (Gaffney et al., 2018). Most sexually active people are infected with HPV during their lifetime, sometimes resulting in genital warts (Herweijer et al., 2018). Severe types of HPV viruses can also result in cancers in cervix, vagina, vulva, pharynx, penis, and anus (Brianti et al., 2017). To eliminate cervical cancer globally, World Health Organization aims for 90% of all girls by 2030 to be fully vaccinated against HPV before they reach 15 years of age (World Health Organization, 2020).

Parents in Sweden trust the national immunization program (NIP) and continued high vaccination rates include 97% of 2-year-old children who are fully and appropriately vaccinated for their age (Public Health Agency of Sweden, 2021a). HPV vaccination was introduced in NIP for girls in 2012; boys were included in August 2020. Vaccine is offered free of charge by School Health Services to children aged 11 to 12 years to prevent cancers later in life (Public Health Agency of Sweden, 2021a). Vaccination coverage in 2021 was one dose each for 87 % of girls and 83 % of boys (Public Health Agency of Sweden, 2021b).

Parents’ knowledge is important in decisions about children’s vaccinations, and it has been associated with increased vaccine uptake (Akmatov et al., 2018). Lack of knowledge, however, has been a reason for parents not to accept HPV vaccination. Understandable and clear information about HPV vaccination has been requested by both girls and parents (Hendry et al., 2013; Holman et al., 2014). Another study suggested that knowledge about HPV and HPV vaccination differs between genders and that girls have more knowledge than boys. A reason for this might be that until recently vaccine only was offered to girls and was therefore seen as a women’s health issue (Patel et al., 2016). In Sweden, school nurses have been found to be both main source of this information (Grandahl, Tydén, et al., 2017; Runngren et al., 2021) and the person who administer vaccine through School Health Services.

When the NIP introduced gender-neutral HPV vaccination, the Public Health Agency of Sweden launched a new information package for children and their parents. The material included an HPV vaccination fact sheet for parents and a short film for children about reasons to get vaccinated against HPV. A PowerPoint presentation was also published for school nurses to use to support their conversations about HPV vaccination with parents and children (Public Health Agency of Sweden, 2022).

## Aim

The aim of this study was to investigate children’s and parents’ attitudes and knowledge about HPV vaccination following the introduction of gender-neutral vaccination and a targeted information intervention in Sweden.

## Materials and methods

### Design

This cross-sectional study with a descriptive design was performed using a web-based questionnaire.

### Population and settings

An invitation letter introducing the study was sent by email to all managers of municipal School Health Services in Sweden (*N =* 290). Each manager who responded allowed the researchers’ access to school nurses in their district. Children who were offered their first dose of HPV vaccine in autumn of 2020 or 2021 and their parents were included in the study, after being informed by the school nurses.

### Data collection

Data were collected from March 2021 to January 2022. The school nurses forwarded the information letter to parents, containing links to the web questionnaires for both children and parents. Anonymous answers through the web-based questionnaires were automatically entered into to a database, ORU-Survey (Sunet Survey/Survey and Report, Artisan Global Media, Växjö, Sweden). Usability and technical functioning were tested by the research group before distributing the final version of the survey. The survey was not password protected, but the link to the survey was only given in the information letter. No system was used to prevent multiple answering from the same person. No other advertising or promoting of the survey was made.

### Questionnaires

Two web-based questionnaires, one for children and one for parents, were designed through scientific discussions within the group of researchers to confirm their relevance and clarity. The questionnaires included a combination of multiple-choice questions, questions to be answered on Likert-type scales (1–10), as well as open-ended questions. The information letter and questionnaires were translated by a professional translator to five different languages: Arabic, English, Somalian, Tigrinya, and Dari. Language selection was based on previous experience of researchers from the Public Health Agency. Parents were asked 37 questions about HPV, HPV vaccination, the information package, and NIP. Most of the multiple-choice and Likert-type questions were followed by an option to provide a free-text answer. For example, the question, ‘Has your child been vaccinated against HPV?’ was followed by an option for those who answered ‘no’ to give a reason. Another question asked whether parents knew which disease or diseases HPV vaccination protects against. The children were asked 14 questions with fixed responses such as ‘Which people are most important to vaccinate against HPV?’ and most such questions were followed by an open-ended question. For example, one open-ended question asked how they perceived HPV information in the film part of the targeted information. Information on sociodemographic variables were also collected (Table 1). All items were presented on the same webpage, with no page breaks. Most of the items requested a mandatory answer but had the option to answer, for example, not applicable or I don’t know.

**Table 1.** Sociodemographic characteristics of the parents (*N* = 276).

	*n* (%)
Parents age, years	
22–32	3 (1)
33–43	134 (49)
44–54	119 (43)
55–65	2 (1)
Missing	18 (7)
Relation to the child	
Father	45 (16)
Mother	230 (83)
Other caregiver	1 (1)
Country of birth	
Sweden	247 (89)
Other European country	16 (6)
Other country outside Europe	13 (5)
Living situation	
Married/living with partner	237 (86)
Partnered but not living together	14 (5)
Single parent living with child	24 (9)
Missing	1 (1)
Educational level	
Compulsory school	3 (1)
High school	50 (18)
University/college	223 (81)

Before the questionnaire was distributed, it was piloted by five parents and their children who had been offered the HPV vaccination. The pilot test was conducted to evaluate feasibility and comprehensibility of the questionnaire. Some minor wordings were adjusted in children’s questionnaire: for example, ‘fact sheets’ were changed to ‘information sheets’.

### Data analysis

Only completed surveys could be submitted, and thus no incomplete answers were analysed. Numerical data were analysed using the SPSS software package (IBM Corp, Released 2021.IBM SPSS Statistics for Windows, Version 28.0. Armonk, NY, USA), and are presented using means, standard deviations (*SD*) after checking for normal distribution, numbers, and percentages when appropriate. The open-ended question in the web survey did not provide enough text to enable a regular content analysis; instead, examples of answers were quoted to highlight results raised by respondents. The selection of quotes was discussed among authors.

## Ethical considerations

Consent for the study was sought from Swedish Ethical Review Authority, which had no ethical objections against the project (registration number: 2020-05336). Questionnaires were answered anonymously by children and parents. By giving their child access to the web questionnaire, parents gave their consent for the child to participate in the study. Only researchers had access to data. No incentives were offered for participating in the study.

## Results

### Characteristics of participants

Twenty-two school managers agreed to send the information letter through school nurses working in that area. A total of 155 schools were included, representing municipalities in both urban and rural areas nation-wide.

As most 275 (99%) of respondents reported being the child’s parent, we refer to all respondents as parents. In total, 276 parents and 206 children responded to the surveys. Mean age of the parents was 43.3 (*SD* 4.6) years. Two-hundred and thirty (83%) parents were mothers, and a majority were born in Sweden (*n* = 247, 89%). Two-hundred and twenty-three (81%) participants had a university or college degree. Sociodemographic characteristics of responding parents are summarized in Table 1.

### Views on HPV vaccination

Two-hundred and twenty-two parents (80%) reported that they felt positively about NIP in general, and they considered vaccinations important for health and prevention of disease (*n = 258,* 93%). According to parents, most children were vaccinated in conformity with NIP guidelines (*n* = 266, 96%). Ninety seven percent (*n =* 269*)* of children were vaccinated against HPV, but six (2%) of the parents did not know whether their children were vaccinated against HPV. Three (1%) of responding parents chose not to vaccinate their child against HPV. One parent reasoned, ‘*There is too little research on boys and the vaccine and future reproduction*’.

### Reasons to vaccinate

Parents mainly vaccinated their children against HPV because they believed the vaccine to be important for their children’s health (Table 2). Parents also perceived that giving HPV vaccination was part of their parental responsibility (*n =* 199, 72%) and equally important for society and health of other individuals (*n* = 158, 5%). Some parents’ reasons to vaccinate their children were expressed in answer to an open-ended question: ‘*I had cervical cancer/cell changes*’ or ‘*I had cervix cancer in my family, therefore this is of great importance*’.

**Table 2.** Reasons for parents to vaccinate their child against HPV.

If you have agreed to your child’s vaccination, whether or not they have had it, or if you expect to do so later, what are your reasons?	Number of answers, *n (%)*^a^
It is important for my child/children’s health	216 (78)
It is my responsibility to my child/children to ensure that they receive vaccination	199 (72)
It is important for the society and the health of other individuals that my child is vaccinated	158 (57)
We were recommended/offered HPV vaccination and therefore we said yes	109 (39)
It’s ‘something you just do’	20 (7)

^a^More than one answer was possible. Percent numbers report the proportion of the 276 respondents who gave that answer.

Both children and parents considered it important to vaccinate all genders against HPV. However, more children than parents thought it was more important to vaccinate girls than boys, whereas parents favoured both genders (OR 1.587, 95% CI 1.085–2.321).

### Questions and concerns about HPV vaccinations

Regardless of vaccination status, 30 (11%) parents reported they felt hesitation and/or concerned making decision to vaccinate their children against HPV, due to lack of good and reliable information about the vaccine (*n =* 14, 47%). Sixteen (5%) parents stated that they had been exposed to negative information about vaccinations in general and 19 (63%) described having concerns about side effects of HPV vaccine.

Even parents who stated that they had knowledge about HPV had some concerns about vaccinating their child. The most common reason for this hesitancy was their fear of potential side effects, as shown in Table 3.

**Table 3.** Reasons to hesitate and/or have concerns about the HPV vaccination.

What were the reasons that you hesitated and/or felt concerned about vaccinating your child against HPV?	Number of answers, *n (%)*^a^
I am worried about the side effects of vaccinations	19 (63)
I lack good and reliable information about the vaccination	14 (47)
I have read or heard negative information about vaccination	5 (17)
I think the risk of being infected by the diseases is small	4 (13)
I want to delay the vaccinations until my child is older	2 (7)
I do not think vaccinations are effective	2 (7)
I am generally hesitant about vaccinations	1 (3)

^a^More than one answer was possible. Percent numbers report the proportion of the 30 respondents in this question who gave that answer.

**Figure 1.**
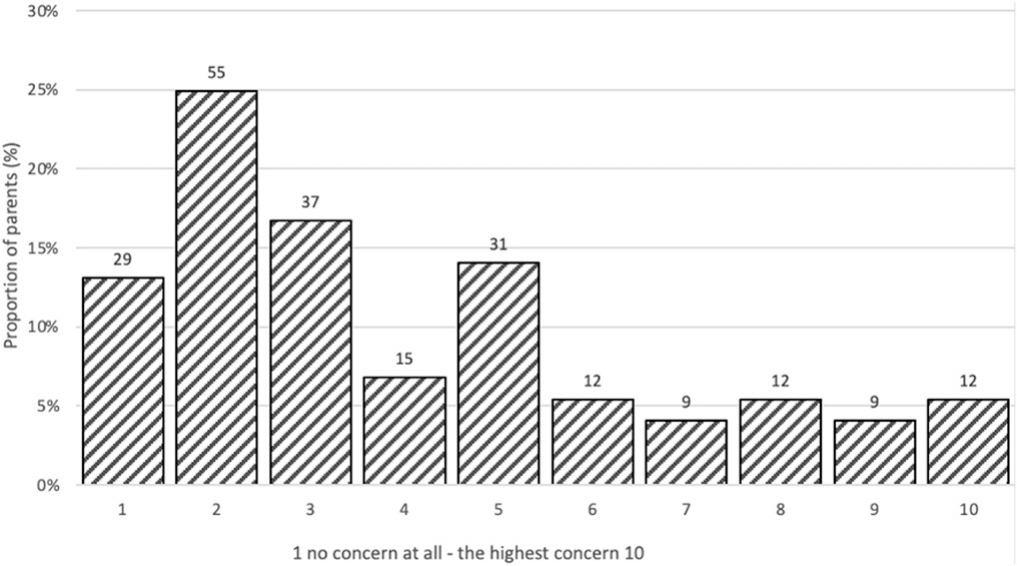
Concerns about side effects from vaccine.

Parents ranked their levels of concern about side effects caused by vaccines in general, as shown in Figure 1, ranging from no concerns (level 1) to highest level of concern (level 10). The variation on the scale, with a higher distribution to left, is notable and shows parents’ concerns regardless of their knowledge about HPV vaccination.

### Knowledge about HPV

Both parents and children were asked if they knew which disease or diseases HPV vaccination protects against in an open-ended question. Parents’ main responses were cervical cancer (*n =* 156, 57%), cancer (*n =* 49, 18%), condyloma (*n =* 20, 7%), and 12 (4%) answered cancer specifically in boys. The most common responses among the children were cancer (*n =* 53, 26%) followed by uterine cancer (*n =* 31, 15%) and cervical cancer (*n =* 28, 14%). Genital cancer was suggested by 9 (4%) of the children.

Some parents commented that they had good knowledge about HPV and HPV vaccination as they worked in health care, for example, as a nurse or physician. Three (1%) parents got the knowledge from their own experience of HPV; as one parent said, ‘*I myself have had HPV18 and undergone conization several times, and most recently I had surgery to remove my uterus*’. Some parents reported that they did not know that boys also were included in the HPV vaccination programme (*n =* 2, 1%).

### Information about HPV vaccinations

Half of the children and two-thirds of the parents had received information about HPV provided by the Public Health Agency. Parents and children had received the information in several different ways, as shown in Table 4.

**Table 4.** Answers to questions about the Public Health Agency’s informational material on HPV vaccination. The result reports the number and proportion in percent of the responding parents and children.

	Parents (*N* = 276), *n* (%)	Children (*N* = 206), *n* (%)
Have you seen the film about HPV vaccination?		
Yes, I have seen the film	21 (8)	40 (19)
The film was: easy to understand/difficult to understand	17 (6)/2 (1)	37 (18)/5 (2)
Have you seen the power point presentation?		
Yes, I have seen the power point presentation	12 (4)	26 (13)
The power point presentation was: easy to understand/difficult to understand	7 (3)/2 (1)	28 (14)/5 (2)
Have you seen the fact sheet?		
Yes, I have seen the fact sheet	153 (55)	42 (20)
The fact sheet was: easy to understand/difficult to understand	123 (45)/1 (1)	47 (23)/22 (11)
No, I have not received any of the information mentioned above in this matter	101 (37)	106 (51)
Did the material from the Public Health Agency lack any information? yes/no	22 (8)/212 (77)	40 (19)/153 (74)

The majority were pleased with information in the material about HPV vaccination. Some parents and children had received information in more than one way. For both parents and children, the fact sheet was most commonly used. However, one child said, ‘*I know I’m going to be vaccinated but I do not know why or what it protects against*’. Three (1%) of children wanted more information about side effects. One child described after having seen the film that ‘*it is good to understand why I should get the vaccination*’, whereas another child described the film as ‘*boring*’.

**Figure 2.**
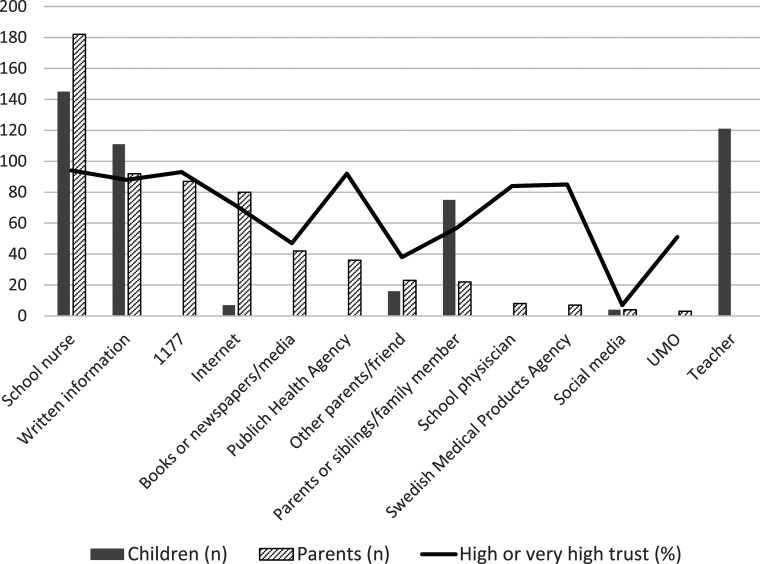
Sources of information for parents and children (number, more than one option was possible, bars). The dark line indicates how many percent of the parents who indicated that they had high or very high trust in each source. 1177 is a medical advice website and UMO stands for a youth guidance centre.

Primary sources from which parents obtained information about HPV vaccination was school nurses (*n =* 182, 66%) followed by written information (*n =* 92, 33%) (Figure 2). Social media (*n =* 4, 1%) and UMO.se (a web site for young persons from 13 to 20 years with information about the body, sex, and health) were used least (*n* = 3, 1%).

Parents rated school nurses (*n =* 260, 94%) as most reliable sources of information about HPV vaccination followed by Medical Advice website 1177.se (*n =* 252, 91%), and Public Health Agency (*n =* 250, 91%). Social media was the source of information about HPV vaccination that parents had least confidence in.

For children, the main source of information about HPV vaccination was school nurses (*n* = 145, 70%) and teachers (*n =* 121, 59%). The internet (*n =* 7, 3%) and social media (*n =* 4, 2%) were shown their least used sources for HPV vaccination information. Books or newspaper-media, 1177.se (official Swedish healthcare website), Public Health Agency, school physicians, and Swedish Medical Products Agency were not reported as sources at all by the children.

Most parents agreed they had received information well enough in advance to make an informed decision about HPV vaccination (*n =* 242, 88%) and that they received information from a reliable source (*n =* 250, 91%). The parents who felt that they received the information too late thought it should have been available before semester started, or a year in advance of vaccination offer, and that information and consent forms should be sent separately. Not all parents were aware that HPV vaccination was offered to boys, and they therefore requested additional information. As two parents stated, ‘*I would like* m*otivational information why boys should be vaccinated*’, and ‘*I had not been reached by information that they now vaccinate boys when we received the vaccination consent from the school*’. One parent requested ‘*an information sheet with pros, cons, and side effects about HPV vaccination from Public Health Agency or Public Health Care*’.

## Discussion

The web-survey, covering children and parents in rural and urban areas of Sweden, has provided a picture of knowledge and attitudes regarding HPV vaccination. This study showed that half of the children and about a third of the parents had received the information package. In Sweden, all parents and children aged 11 to 12 years are informed about HPV vaccination by school nurses, confirmed by this study as school nurses were most commonly reported source of information about HPV. Previous studies have also shown that school nurses are an important source of HPV and HPV vaccination information (Grandahl, Larsson, Tydén, & Stenhammar, 2017; Runngren et al., 2021). To our knowledge, this is the first survey to investigate attitudes and knowledge of children and parents regarding HPV vaccination after receiving the information package from the Swedish Public Health Agency. The package was launched in spring 2020 just before boys were included in NIP. This study, like a previous study (Runngren et al., 2021), shows continued trust in the Swedish vaccination program. Even if parents felt some concern about possible side effects of the vaccine, they still chose to vaccinate their children. Another study reported side effects of a vaccine to be the most common concern, especially when the vaccine was new (Karafillakis & Larson, 2017).

Where teachers get information about HPV and HPV vaccination, they give to children in school is also of interest, as children should receive evidence-based information. This might be problematic as studies have shown that teachers often lack real knowledge about HPV and HPV vaccination (Keten et al., 2021; Masika et al., 2015). Knowledge about HPV also differs between female and male teachers, with female teachers demonstrating greater HPV knowledge than male teachers (Keten et al., 2021). It was also shown in a Swedish study that school nurses and teachers did not cooperate in providing information to students about HPV (Odenbring and Lindén, 2022). As teachers are an important source of information for children, they need to have adequate knowledge about vaccinations (Siu et al., 2019). Cooperation between school nurses and teachers could ensure that parents and children receive the same information. To facilitate provision of good information to children, teachers would also benefit from an information package use when information about HPV and HPV vaccination is given to children at school.

It has been shown in previous research that information is an important part of parents’ informed decisions (Hendry et al., 2013). In the present study, about one third of the parents and about half of the children had not seen any information material at all from the Public Health Agency. Although several children and parents had not seen the material, this lack had no great effect on their actions. In total, 87% of the girls and 83% of the boys were vaccinated with a first dose of HPV vaccine in 2020 (Public Health Agency of Sweden, 2021a). This might indicate a continued high level of trust in NIP. This is also supported in a study in which parents described high levels of trust in both vaccine recommendations and health care professionals (Bystrom et al., 2020).

To make decisions, children and parents need the ability to acquire, understand, and use information in order to maintain, promote, or improve health, which can be described as health literacy (Nutbeam et al., 2018). Health literacy effects people’s health outcomes (Kickbusch, 2001) and is necessary for them to make good health decisions and have control over their own health. This includes making informed decisions about HPV vaccination. As described in the results, knowledge about HPV differs among parents and low or limited health literacy is a risk factor for poorer health outcomes for both children and adults (Vaillancourt and Cameron, 2022). Health literacy changes over a person’s lifetime and is often linked to socioeconomic circumstances such as gender and education (Vaillancourt and Cameron, 2022). Raising awareness of the importance of health and how to maintain health in young people’s lives can provide conditions for good health in adulthood (World Health Organization, 2023). By understanding young people’s health in their social context, society has opportunities to give young people conditions for good lifestyle habits. Therefore, it is important to increase people’s early health literacy, especially regarding HPV and sexual health.

Social media can provide both positive and negative information, but they are not always reliable about HPV and HPV vaccination (Ortiz et al., 2019). We found in this study that social media was one of the least used sources of information for both children and parents. Although vaccines are described on several social media platforms (Puri et al., 2020) and the information is often intended and used in terms of health literacy (Chen et al., 2018), the percentage of social media use in this study was low. It has also been shown that people with low health literacy more commonly use social media for information (Chen et al., 2018), which may say something positive about health literacy in our study population. Health is a prerequisite for children to be able to acquire knowledge, and one goal for schools in their health promotion work is to strengthen young people’s well-being and draw attention to health and lifestyle issues (Swedish National Agency for Education, 2022). By organizing for collaboration between School Health Services and teachers, it can help create environments that promote health literacy and health for the children.

## Strengths and limitations

This study presents data from children and parents who had been offered first dose of HPV vaccination, over a broad geographical area. A strength of the open-ended questions was that the participants had an opportunity to express themselves freely, which increases trustworthiness of results (Lincoln, 1985). As described, we did not make any qualitative analysis of the open-ended answers, but after discussion in the research group chose answers that were representative to illustrate the different questions. Translation of the questionnaires into several languages was another strength, as it provided us an opportunity to include as many participants as possible and to obtain different perspectives on HPV.

A limitation is lack of information regarding children and parents who did not respond to the web survey, as there may be important differences between those who participated in the study and those who chose not to. It is not uncommon for a nonresponse bias to occur in cross-sectional studies (Wang and Cheng, 2020). Time between the information and the vaccination occasion also differed among the participants; therefore, there is a risk for recall bias in this study.

The method of distributing the survey via the school health organizations and school nurses makes it impossible to report response rate or representability of answering persons, but the fact that participating schools were distributed over both rural and urban areas speaks in favour of a representative sample.

## Implications for practice

Vaccinations are a concrete way of preventing disease (Castle and Maza, 2016). Because teachers are an important source of information for children, teachers might require increased knowledge about HPV (Keten et al., 2021; Masika et al., 2015). The school nurses and teachers could work together with common information and ensure that parents and children receive the same knowledge. Another option is to provide an information package to support teachers when information about HPV and HPV vaccination is given to children at school. Further research should focus on teachers and their knowledge of HPV. It is necessary to explore further how parents and children use social media when searching for health information and how authorities can use social media as a tool in their work to increase health literacy and increase compliance with immunization programs among the population.

## Conclusion

Parents highlight the importance of vaccinations and describe HPV vaccination as important for their children’s health, the society, and health of other individuals. For parents to make an informed decision about vaccination, information is necessary. In this study, half of the children and about a third of the parents were reached with the information package about HPV vaccination, which can be seen as an opportunity for improvement. However, the vaccination coverage was still high, which confirms continued trust in NIP in Sweden.

This study showed that the school nurse is the most commonly used and trusted source of information about HPV; teacher was also a common source of information for children.

## Supplemental Material

Supplemental Material - Children’s and parents’ attitudes to and knowledge about HPV vaccination following a targeted information intervention Supplemental Material for Children’s and parents’ attitudes to and knowledge about HPV vaccination following a targeted information intervention by Eva Runngren, Karin Blomberg, Lina Schollin Ask, Emma Appelqvist, Madelene Danielsson, and Mats Eriksson in Journal of Child Health Care.
