# Supplementary material for: Children’s and parents’ attitudes to and knowledge about HPV vaccination following a targeted information intervention
Source: J Child Health Care. 2024 Sep 27;29(4):962–74. doi: 10.1177/13674935241272004 (PMC12662824; doi:10.1177/13674935241272004)
Supplement: Supplemental Material - Children’s and parents’ attitudes to and knowledge about HPV vaccination following a targeted information intervention [file sj-pdf-1-chc-10.1177_13674935241272004.pdf]

HPV vaccination Questionnaire – Parents and guardians

Participation in the study is voluntary and by submitting the completed questionnaire you grant us permission to use your answers. The answers will be processed without revealing your or your child's identity. The questions refer to the child/children who was/were recently offered an HPV vaccination through the school health service. From now on we will use the term "child" even if there are several children in your household who have been offered an HPV vaccination.

My relationship to the child:

☐ Mother

☐ Father

☐ Other guardian

The child/children in the family who was/were offered an HPV vaccination in autumn 2020:

☐ Girl

☐ Boy

☐ Several children, both boys

☐ Several children, both girls

☐ Several chrildren, different genders

☐ Other

☐ We/my child have/has not been offered an HPV vaccination

My age:

[ ]

My country of birth:

☐ Born in Sweden

☐ Born in another country in Europe

☐ Born in another country outside Europe

I am:

☐ Married/cohabiting

☐ Non-cohabiting partner

☐ Single (including widow/widower)

Number of persons in the household:

[ ]

Apart from the child/children who has/have been offered the HPV vaccine, state the age of the other children in the household: If there are more than five children in addition to the child/children who has/have been offered the HPV vaccine, provide an answer for the five oldest

Child 1, age: ____________

Child 2, age: ____________

Child 3, age: ____________

Child 4, age: ____________

Child 5, age: ____________

What is your highest completed level of education? If you are still studying, choose your highest completed level of education to date.

- ☐ Secondary/primary school, elementary school, junior-secondary school, or similar
- ☐ 2-year high school or vocational college
- ☐ 3/4-year high school
- ☐ Folk high school or similar
- ☐ University or college, less than 3 years
- ☐ University or college, 3 years or more

Has your child been vaccinated against HPV?

- ☐ Yes
- ☐ No
- ☐ Don’t know
- ☐ I don’t wish to answer

Why has your child not been vaccinated against HPV?

- ☐ I/we chose to decline the vaccination
- ☐ I/we missed the child’s vaccination appointment
- ☐ I/we were not summoned for or offered a vaccination
- ☐ Don’t know
- ☐ Other
- ☐ I don’t wish to answer

 o If other, please specify:

If you have chosen not to have your child vaccinated against HPV, what was the reason? You may choose more than one answer

- I don’t believe the disease is dangerous ☐
- I don’t believe a vaccination is necessary ☐
- I believe there is a low risk of being infected with the disease ☐
- I don’t have good, reliable information about the vaccination ☐
- I have read or heard negative things about the vaccination ☐
- I don’t believe vaccinations are an effective way of preventing disease ☐
- I’m concerned about the risk of side-effects from the vaccination ☐
- I want to wait with vaccinations until the child is older ☐
- I’m generally doubtful about vaccinations ☐
- Don’t know ☐
- I don’t wish to answer ☐

 Other:

If you have declined the offer of a vaccination against HPV, will you accept later?

- ☐ Yes
- ☐ No
- ☐ Don’t know
- ☐ I don’t wish to answer

If your child has had an HPV vaccination, you have agreed to an HPV vaccination, or if you expect to do so later, what is the reason? You may choose more than one answer

- ☐ I have a responsibility to my child/children to ensure they are vaccinated
- ☐ It is important for the community and the health of other individuals that my child is vaccinated
- ☐ It is important for my child’s/children’s health
- ☐ We were recommended/offered HPV vaccination and that is why we accepted
- ☐ It is “just something you do”
- ☐ I don’t wish to answer

 Other:

Were you doubtful or did you feel worried before your child was vaccinated against HPV?

- ☐ Yes, I was worried
- ☐ Yes, I was doubtful
- ☐ Yes, I was both doubtful and worried
- ☐ No
- ☐ Don’t know
- ☐ I don’t wish to answer

What is the reason why you were doubtful and/or worried before your child was vaccinated against HPV? You may choose more than one answer

☐ I don’t have good, reliable information about the vaccination

☐ I have read or heard negative things about the vaccination

☐ I don’t believe the disease is dangerous

☐ I believe there is a low risk of being infected by the disease

☐ I don’t believe that vaccinations are effective

☐ I’m worried about the risk of side-effects from the vaccination

☐ I want to wait with the vaccinations until the child is older

☐ I’m generally doubtful about vaccinations

☐ Don’t know

☐ I don’t wish to answer

 Other:

Do you know any other diseases the HPV vaccination protects against?

☐ Yes (write down which diseases on the comment line)

☐ No

 Comment

Which group is it most important to vaccinate against HPV?

☐ Girls

☐ Boys

☐ Equally important

☐ Neither of them

When you received or obtained information about the HPV vaccination for your child, where did you get the information from? You may choose more than one answer. Mark all the answers that are relevant to you.

S- ch- oo- l nu- rs- e	S- ch- oo- l do- ct- or	I- nf- or- m- ati- on sh- ee- t or fa- ct sh- ee- t	F- a- mi- ly m- e- m- be- r or fri- en- d	T- al- ki- ng to ot- he- r pa- re- nt- s/- gu- ar- di- an- s	P- ub- lic H- ea- lth A- ge- nc- y	1- 17- 7 H- ea- lth- ca- re G- ui- de (H- ea- lth- ca- re A- dv- ic- e Li- ne-)	- M- ed- ic- al Pr- od- uc- ts A- ge- nc- y	- U- M- O.- se (Y- ou- ng Pe- rs- on- s’ Cl- ini- c O- nli- ne-)	- M- ed- ia (T- V, ra- di- o, ne- ws- pa- pe- rs)	P- er- so- na- l inf- or- m- ati- on se- ar- ch on the Int- er- ne- t	S- oc- ial m- ed- ia (F- ac- eb- oo- k, T- wi- tte- r, In- sta- gr- a- m et- c.)
☐	☐	☐	☐	☐	☐	☐	☐	☐	☐	☐	☐

 Other:

How confident are you about the following sources in terms of information about vaccinations for your child?

	Very confident	Quite confident	Slightly confident	Not confident	No opinion	I don't wish to answer
School nurse	☐	☐	☐	☐	☐	☐
School doctor	☐	☐	☐	☐	☐	☐
Information sheet or fact sheet	☐	☐	☐	☐	☐	☐
Family member or friend	☐	☐	☐	☐	☐	☐
Talking to other parents/guardians	☐	☐	☐	☐	☐	☐
Public Health Agency	☐	☐	☐	☐	☐	☐
1177 Healthcare Guide (Healthcare Advice Line)	☐	☐	☐	☐	☐	☐
Medical Products Agency	☐	☐	☐	☐	☐	☐
UMO.se (Young Persons' Clinic Online)	☐	☐	☐	☐	☐	☐
Media (TV, radio, newspapers)	☐	☐	☐	☐	☐	☐
Personal information search on the Internet	☐	☐	☐	☐	☐	☐
Social media (Facebook, Twitter, Instagram etc.)	☐	☐	☐	☐	☐	☐

Do you feel you received information in the right way/via the right channels?

☐ Yes

☐ No

☐ Don't know

If not, how would you like to receive the information?

When you received information about the HPV vaccination did you feel you had enough time to think and make a decision about the vaccination?

☐ Yes

☐ No

☐ Don't know

If not, when would you have preferred to receive the information?

Have you seen or read the following information material from the Public Health Agency? You may choose more than one answer.

Yes, I have seen information films about the HPV vaccination	☐
Yes, I have received verbal information from the school nurse or school doctor when they spoke about HPV with the use of pictures	☐
Yes, I have received a fact sheet entitled "Useful information about HPV"	☐
No, I have not had access to any of the above information on the subject	☐

What did you think about the film? Write a number from 1 = very bad to 10 = very good

Easy to understand	
Difficult to understand	
I have not seen the film	

Opinion about the film:

What did you think about the discussion with the school nurse with the use of pictures? Write a number from 1 = very bad to 10 = very good The discussion and pictures were:

Easy to understand

Difficult to understand

Mark with a cross if you have not taken part in a discussion or seen the pictures

 Opinion about the discussion with the school nurse with the use of pictures:

What did you think about the fact sheet? Write a number from 1 = very bad to 10 = very good The fact sheet was:

Easy to understand

Difficult to understand

Mark with a cross if you have not seen/read the fact sheet

 Opinion about the fact sheet:

Did you feel there was anything missing from the information material from the Public Health Agency?

☐ Yes

☐ No

 If you answered Yes, what did you feel was missing?

What is your general standpoint with regard to vaccinations for children?

  I don’t wish to answer (mark with a cross)

How important do you feel vaccinations are generally for your child’s/children’s future health?

I don’t wish to answer (mark with a cross)

How concerned are you generally about the side-effects of vaccinations for children?

 I don’t wish to answer (mark with a cross)

Has your child received all the vaccinations offered by the child welfare centre or school health system in Sweden?

- ☐ Yes
- ☐ Yes, but not all
- ☐ No
- ☐ Don’t know
- ☐ I don’t wish to answer

If your child/children has/have received the vaccinations as part of the vaccination programme, what was the reason? You may choose more than one answer.

I have a responsibility to my child/children to ensure they are vaccinated	☐
It is important to the community and the health of other individuals that my child is vaccinated	☐
It is important for the health of my child/children	☐
We were recommended/offered a vaccination and that is why we accepted	☐
It is “just something you do”	☐
I don’t wish to answer	☐

 Other:

Why has your child not received all the vaccinations that have been offered?

- ☐ I/we chose to decline the vaccination(s)
- ☐ I/we missed the child’s vaccination(s)
- ☐ Don’t know
- ☐ I don’t wish to answer
- ☐ Other:

What is the reason why you declined a vaccine that was offered free of charge in Sweden? You may choose more than one answer.

- [ ] I don’t believe the disease is dangerous
- [ ] I don’t believe vaccination is necessary
- [ ] I believe the risk of being infected with disease is low
- [ ] I don’t have good, reliable information about the vaccination
- [ ] I have read or heard negative things about the vaccination
- [ ] I don’t believe vaccinations are effective
- [ ] I’m concerned about the risk of side-effects from the vaccination
- [ ] I want to wait with the vaccinations until the child is a little older
- [ ] I’m generally doubtful about vaccinations
- [ ] I/we did not keep the appointment at the child welfare centre/school health service for practical reasons
- [ ] I/we were not summoned for or offered a vaccination
- [ ] Don’t know
- [ ] I don’t wish to answer

 Other:

Have you at any time felt doubtful or worried before vaccinating your child?

- ☐ Yes, worried
- ☐ Yes, doubtful
- ☐ Yes, both doubtful and worried
- ☐ No
- ☐ Don’t know
- ☐ I don’t wish to answer

What is the reason why you were doubtful and/or worried before having your child vaccinated? You may choose more than one answer.

I don’t have good, reliable information about the vaccination	☐
I have read or heard negative things about the vaccination	☐
I don’t believe the disease is dangerous	☐
I think the risk of being infected with disease is low	☐
I don’t believe vaccinations are effective	☐
I’m worried about the risk of side-effects from the vaccination	☐
I want to wait with the vaccinations until the child is a little older	☐
I’m generally doubtful about vaccinations	☐
Don’t know	☐
I don’t wish to answer	☐

 Other:

☐ Ye
☐ s
No

 Comment:

You can also contact us by email at eva.runngren@oru.se Thank you for taking part!

HPV vaccination, Pupil questionnaire

Taking part in the study is voluntary and by submitting the questionnaire you give us permission to use your answers. No one will be able to see that it is you who has answered.

I am:

☐ Girl
☐ Boy
☐ Other/I don’t wish to answer

What year are you in?

☐ Year 5
☐ Year 6

Have you been vaccinated against HPV?

☐ Yes
☐ No
☐ I have not been offered a vaccination
☐ Don’t know

Do you know any other disease or diseases the HPV vaccination protects against?

☐ No
☐ Yes (write which disease/diseases):

Which group is it most important to vaccinate against HPV?

☐ Girls
☐ Boys

☐ Both are equally important

☐ Non of them

How did you receive information about the HPV vaccination? You may choose more than oneanswer.

The school nurse told me	☐
My teacher told me	☐
I received a letter or a paper with information	☐
I read about it in a textbook or a newspaper	☐
I read about it on the Internet	☐
I read about it on social media	☐
My parents or siblings told me	☐
My friends told me	☐
I have not received any information	☐

Other:

Have you seen the following information material? You may choose more than one answer.

I have seen an information film about HPV vaccination	☐
I have talked to the school nurse with the use of pictures	☐
I have received a fact sheet entitled “Useful information about HPV”	☐
I have read about it on the 1177 website	☐
I have read about it on the Young Person’s Clinic website	☐
I have read about it on the Public Health Agency website	☐
I have not seen any information	☐

 Other:

Have you seen the following information material from the Public Health Agency? You may choose more than one answer.

Yes, I have seen an information film about HPV vaccination	☐
Yes, I have received information from the school nurse or the school doctor when they spoke about HPV with the use of pictures	☐
Yes, I have received a fact sheet entitled "Useful information about HPV"	☐
No, I have not received any information	☐

What did you think about the film? (Write a number from 1 = very bad to 10 = very good) Thefilm was:

Easy to understand	
Difficult to understand	
I have not seen the film	

Here you can write what you thought about the film:

What did you think about the discussion with the use of pictures that you had with the schoolnurse or school doctor? (Write a number from 1 = very bad to 10 = very good)

Easy to understand

Difficult to understand

I have not had any discussion or seen any pictures

Here you can write here what you thought about the discussion with the school nurse using pictures:

What did you think about the fact sheet? (Write a number from 1 = very bad to 10 = very good)The fact sheet was:

Easy to understand

Difficult to understand

I have not seen the fact sheet

Here you can write what you thought about the fact sheet:

Was there anything missing from the information?

☐ No

☐ Yes

 Here you can write what you thought was missing from the information:

Do you have any comments about this questionnaire, or do you have anything to add?

Do you have any questions about the HPV vaccination that are still unanswered?

☐ Yes

☐ No

Here you can write your question/questions about the HPV vaccination:

Do you wish to say anything about this questionnaire or about HPV vaccination?

☐ Yes

☐ No

If you have something you wish to say about the questionnaire/HPV vaccination, you can write it here:

You can also email us at eva.runngren@oru.se Thank you for taking part!
